# Grey matter volume and fractional anisotropy as biomarkers of cognitive change in traumatic brain injury over a 6-month period

**DOI:** 10.3389/fneur.2025.1602173

**Published:** 2025-10-10

**Authors:** Ben Zhang, Niko Fullmer, Sarah Dunn, Zhong Sheng Zheng, Caroline Schnakers, Emily R. Rosario

**Affiliations:** 1 Research Institute, Casa Colina Hospital and Centers for Healthcare, Pomona, CA, United States; 2 Department of Kinesiology, California State University, San Bernardino, Palm Desert Campus, Palm Desert, CA, United States

**Keywords:** TBI, MRI, DTI, biomarkers, cognitive recovery, traumatic brain injury, fractional anisotropy, RCI

## Abstract

In this study we explored how neuroimaging biomarkers relate to cognitive recovery in traumatic brain injury (TBI) patients. Sixteen participants with moderate to severe traumatic brain injury were enrolled with MRI and diffusion tensor imaging (DTI) collected at enrollment and 6 months. The Repeatable Battery for the Assessment of Neuropsychological Status (RBANS), Disability Rating Scale, and Montreal Cognitive Assessment (MoCA) were also administered at both time points to evaluate neuropsychological and functional outcomes. Composite RBANS score showed significant increase from study enrollment to 6 month follow up (*p* = 0.035). Fractional anisotropy (FA) in the genu (r_s_ = 0.811, *p* = 0.004) and splenium of the corpus callosum (r_s_ = 0.744, *p* = 0.009) was strongly correlated with changes in the RBANS—Attention index score. Left tapetum FA was correlated with changes in the RBANS—Visuospatial/Constructional index score (r_s_ = 0.744, *p* = 0.011). Left temporal fusiform cortex grey matter (GM) volume was correlated with changes in the RBANS—Attention index score (r_s_ = 0.756, *p* = 0.011). Imaging markers measured at the beginning of the study, such as FA and GM volume, are correlated with 6-month cognitive recovery in TBI, supporting the potential use of neuroimaging to guide rehabilitation strategies.

## Introduction

Traumatic brain injury (TBI) is a major cause of long-term disability globally, often resulting in persistent impairments across cognitive domains such as attention, memory, executive function, and processing speed. Neuropsychological assessments such as the Repeatable Battery for the Assessment of Neuropsychological Status (RBANS) and Montreal Cognitive Assessment (MoCA) have been widely used to track cognitive recovery, providing objective measures of improvement over time (1). Due to the significant variability in individual recovery, identifying reliable biomarkers to predict recovery trajectories has become a critical focus of research. Changes in brain structure and function following injury are often associated with neuroplasticity, the brain’s ability to reorganize and adapt through structural modifications and neural network reconfiguration (2, 3). Understanding which structural and biological factors, such as brain volume changes and molecular markers, are associated with neuroplasticity and cognitive recovery is key to improving prognoses and guiding personalized rehabilitation strategies, ultimately enhancing quality of life for TBI survivors (4).

Patients often undergo routine MRI scans as part of their initial evaluation after being admitted to the hospital following the acute phase of TBI (5). These scans provide high-resolution T1-weighted images, allowing for the assessment of grey matter volume. Additionally, diffusion tensor imaging (DTI) can measure integrity of white matter tracts, which are crucial for communication between different brain regions and are particularly susceptible to injury during traumatic events (6). Using DTI metrics such as fractional anisotropy (FA) we are able to obtain detailed information regarding their microstructural integrity. FA is a scalar value that quantifies the directional coherence of water diffusion within white matter. Values range from 0 to 1, where higher values indicate greater white matter integrity, reflecting more organized and structurally intact fiber tracts, while lower values suggest damage or degeneration (7). Computing FA, alongside measurements of grey matter volumes in regions of the cerebral cortex, allows for the identification of potentially significant correlates of cognitive recovery (7).

Extensive research has identified the temporal lobes and corpus callosum as key regions of interest in TBI due to their susceptibility to injury and critical role in cognitive function (8–14). Temporal lobe damage, including contusions in TBI, has been linked to poorer six-month functional outcomes, while dynamic imaging studies have detected medial temporal abnormalities in patients with persistent post concussive symptoms, suggesting a role in chronic memory impairment (15, 16). Similarly, DTI studies highlight corpus callosum disruption, particularly in the genu and splenium, as a common finding in mild to moderate TBI, with reduced white matter integrity in these regions correlating with poorer memory and attention performance (17). Meta-analyses further emphasize the role of both interhemispheric and temporal white matter pathways, such as the uncinate fasciculus, in cognitive recovery (18). These findings reinforce the importance of examining temporal and interhemispheric white matter integrity as potential biomarkers of cognitive outcomes in TBI.

This study aims to explore the prognostic value of neuroimaging markers in predicting cognitive recovery in TBI patients over a 6 month period. By analyzing correlations between initial (at study enrollment) white matter integrity, grey matter volume, and cognitive changes (over 6 months), we aim to identify biomarkers that are reliably associated with recovery trajectories. Early recognition of patients at risk for poorer outcomes could significantly improve the management and rehabilitation of their treatment, and ultimately result in better long-term recovery.

## Methods

### Participants

This study included 16 participants (mean age at time of injury: 41.02 years, SD = 13.83) with moderate to severe TBI who were previously enrolled from an acute rehabilitation hospital setting (mean time since injury: 22.27 months, SD = 39.74). One participant did not complete neurocognitive testing or an MRI scan and was excluded from analyses, resulting in a cohort of 15 participants (12 males and 3 females). Inclusion criteria required participants to have a diagnosis of moderate to severe TBI, be fluent English speakers, have intact motor use of their dominant hand, and have no receptive or expressive language impairments, including aphasia. TBI severity was defined by the Glasgow Coma Scale (GCS). A GCS score of 3–8 was considered severe, and a GCS score of 9–12 was considered moderate. Participants were excluded if they were under the age of 18 or over the age of 65 years. This age limitation was implemented to avoid potential confounding effects of age-related cognitive decline that could occur over a long follow-up period, as significant brain changes could occur in individuals aged 65 + over longer time frames (19). Although the original study aimed for a 2-year follow-up, high participant attrition—especially at the 1-year+ time points—led to a decision to limit analysis to a 6-month time frame to maintain a better sample size.

Additionally, individuals who had claustrophobia or metallic implants that would make MRI assessments unsafe were also excluded from the study. Participants were required to demonstrate an understanding of the study purpose, procedures, and potential risks before providing written consent. In cases where a participant lacked the cognitive capacity to consent, a legally authorized representative—typically a spouse or close family member—provided consent on their behalf. Participants were either screened directly by the research team, who introduced the study to them, or were referred by their clinical team based on eligibility criteria. The average Disability Rating Scale (DRS) score at baseline was 3.67 (SD = 2.58), reflecting a range of functional impairments within the sample. Participants did not receive any experimental or study-specific interventions between the acute phase of injury and the initial study measurement. All individuals were managed with normal and customary clinical care, and at the time of enrollment they were undergoing standard rehabilitation. Demographic and clinical data of the enrolled participants are detailed in Table 1.

**Table 1:** Participant demographics.

Variable	Mean (SD)
Age at Time of Injury	41.02 years (13.83)
Months Post Injury	22.27 (39.74)
Male/Female	12/3
Education (High School/Some College/Associates/Bachelors/Masters+)	5/2/2/4/2
Hispanic/Non-Hispanic	3/12
TBI Severity (Moderate/Severe)	7/8

Radiological reports revealed heterogeneous injury patterns across the cohort, reflecting the variable nature of moderate-to-severe TBI. Frontal and temporal lesions were most common, with frequent involvement of the corpus callosum and occasional thalamic abnormalities. Several participants demonstrated imaging features consistent with diffuse axonal injury, while others showed post-traumatic encephalomalacia or ventricular enlargement. This heterogeneity precluded meaningful subgrouping by lesion location, but it reflects the real-world variability of TBI.

The study was conducted following approval from the Institutional Review Board at Casa Colina Hospital (IRB case number: IRB00002372). All participants were recruited from the Acute Rehabilitation Center or Transitional Living Center at Casa Colina Hospital and Centers for Healthcare, and provided written informed consent prior to their inclusion in the study.

### MRI acquisition

MRI data was acquired using a 3 T Siemens Magnetom Verio scanner. A T1-weighted Magnetization Prepared Rapid Gradient Echo (MPRAGE) sequence was used for the high resolution 3-D anatomical scan. It had the following parameters: repetition time (TR) of 2,300 ms, echo time (TE) of 2 ms, a flip angle of 9°, a field of view (FOV) of 230 mm, and a slice thickness of 1 mm without any gaps. The scan included 160 slices with a matrix size of 224 × 224. Diffusion-weighted imaging (DWI) was conducted with the following settings: TR of 10,900 ms, TE of 95 ms, slice thickness of 2 mm with no gaps, 82 slices, and a matrix size of 122 × 122. Diffusion-sensitizing gradients were applied in 64 different non-collinear directions with a b-value of 1,000 s/mm^2^. Additionally, five images without diffusion weighting (b = 0) were captured.

### T1/DTI preprocessing and analysis

First, T1-weighted images underwent bias-field correction using FSL tools (available at http://www.fmrib.ox.ac.uk/fsl). Brain extraction was then performed using the Optimized Brain Extraction for Pathological Brains (optiBET) technique, and grey matter segmentation was carried out with FMRIB’s Automated Segmentation Tool (FAST) (20). We took a region of interest (ROI) approach with labels from the Harvard-Oxford cortical structural atlas (available at https://neurovault.org/collections/262/), focusing on temporo-cortical regions given the substantial evidence highlighting the relationship between TBI severity and the temporal lobe (8, 9, 12–16).

**Figure 1:**
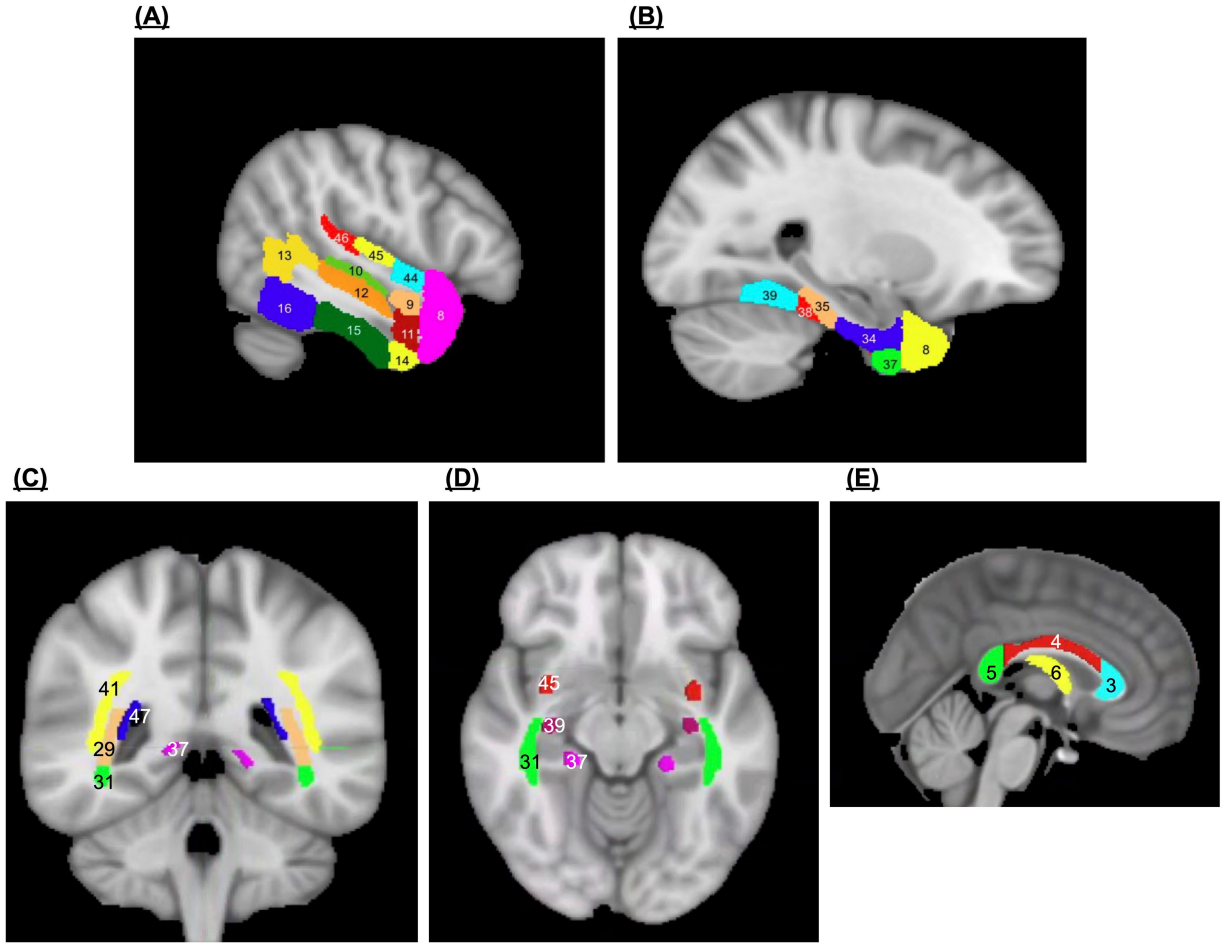
Regions of interest. **(A,B)** Temporal Cortical Grey Matter Regions of Interest: [8] temporal pole, [9] superior temporal gyrus—anterior division, [10] superior temporal gyrus—posterior division, [11] middle temporal gyrus—anterior division, [12] middle temporal gyrus—posterior division, [13] middle temporal gyrus—temporooccipital part, [14] inferior temporal gyrus—anterior division, [15] inferior temporal gyrus—posterior division, [16] inferior temporal gyrus—temporooccipital art, [34] parahippocampal gyrus—anterior division, [35] parahippocampal gyrus—posterior division, [37] temporal fusiform cortex—anterior division, [38] temporal fusiform cortex—posterior division, [39] temporal occipital fusiform cortex, [44] planum polare, [45] Heschl’s gyrus, [46] planum temporale. **(C,D)** Temporal White Matter Regions of Interest: [29] posterior thalamic radiation, [31] sagittal Stratum, [37] cingulum (hippocampus), [39] stria terminalis, [41] superior longitudinal fasciculus, [45] uncinate fasciculus, [47] tapetum. **(E)** Corpus Callosum and Fornix White Matter Regions of Interest: [3] genu of corpus callosum, [4] body of corpus callosum, [5] splenium of corpus callosum, [6] fornix.

Seventeen ROIs were selected (Figure 1): temporal pole, anterior and posterior divisions of the superior temporal gyrus, anterior and posterior divisions of the middle temporal gyrus, temporooccipital middle temporal gyrus, anterior and posterior divisions of the inferior temporal gyrus, temporooccipital inferior temporal gyrus, anterior and posterior divisions of the parahippocampal gyrus, anterior and posterior divisions of the temporal fusiform cortex, temporal occipital fusiform cortex, planum polare, heschl’s gyrus, and planum temporale.

In order to align the cortical ROIs with the patients’ structural data, we performed nonlinear registration between the standard MNI space and each patient’s structural space. Grey matter volume was then calculated for each ROI after they were visually inspected for accuracy. All T1 datasets were retained.

For preprocessing of DWI data, we utilized DTIPrep^1^ to identify and remove volumes with artifacts. This step included corrections for eddy currents and head motion through affine registration to the mean b = 0 reference image. FSL’s Brain Extraction Tool (BET) was then used to strip the skull from the b = 0 image. At each voxel in the brain, diffusion tensors were computed using the diffusion toolbox in FSL, and FA maps were generated. We applied an ROI approach to this as well, using labels from the John Hopkins University (JHU) white matter atlas (available at https://neurovault.org/collections/264/).

Alongside the relationship with the temporal lobe, literature relating to DTI suggests a strong connection between interhemispheric white matter and cognition following TBI. Hence, we opted to examine not only temporal white matter tracts, but those of the corpus callosum as well (10, 11, 17, 18). The following supratentorial ROIs were selected (Figure 1): uncinate fasciculus, cingulum, sagittal stratum, superior longitudinal fasciculus, stria terminalis, fornix, posterior thalamic radiation, genu of corpus callosum, body of corpus callosum, and splenium of corpus callosum, and tapetum.

A similar method to the T1 data was used to align the standard white matter tract ROIs with the patients’ diffusion data. Nonlinear registration between the standard MNI space (FMRIB58_FA) and each patient’s diffusion space was done, then each ROI was visually inspected for accuracy and manually corrected if necessary before data analysis. To ensure that only white matter voxels were included, a whole-brain white matter segmentation mask was generated for each patient using the processed T1-weighted image and then intersected with the ROIs. Finally, the average FA value for each ROI was calculated for each patient. All DTI datasets were retained.

Visual inspection of was done at each step of the brain/atlas registration pipeline for both T1 and DTI data, as well as on the final outputs to ensure that ROIs were aligned for analyses in both native structural and diffusion spaces.

### Procedure/cognitive assessments

We evaluated the recovery trajectory of TBI patients over a six-month period, with data collected immediately following study enrollment, and then after 6 months during dedicated one-week assessment periods. During each of these assessment weeks participants underwent a series of structured evaluations which were designed to provide a comprehensive overview of their recovery progress. Demographic data was collected for each participant at the beginning of their enrollment in the study.

Evaluations conducted at each interval included neuropsychological assessments and magnetic resonance imaging. Neurocognitive assessments were conducted within the same session by trained clinicians. To minimize practice effects, alternate forms of the RBANS were administered at each time point. MRI scans were conducted in a separate session during the same assessment week. The following neuropsychological evaluations were administered by trained clinicians: Montreal Cognitive Assessment (MoCA), consisting of 30 total points: visuospatial abilities (5 points), confrontation naming (3 points), short term memory (5 points), attention (6 points), language (3 points), abstraction (2 points), and orientation (6 points). The Repeatable Battery for the Assessment of Neuropsychological Status (RBANS), with an index score range of 40–160, contained list learning (40 points), story memory (24 points), figure copying (20 points), line orientation (20 points), picture naming (10 points), semantic fluency (40 points), digit span (16 points), list recall (10 points), list recognition (20 points), story recall (12 points), and figure recall (20 points). Raw RBANS scores were converted to standardized index scores, derived from the RBANS manual. The Disability Rating Scale (DRS) has a maximum score of 29, correlating with an extreme vegetative state—a person without disability would have a score of 0. It measures eye opening (3 points), communication ability (4 points), motor response (5 points), feeding awareness (3 points), toileting awareness (3 points), grooming awareness (3 points), level of functioning (5 points), employability (3 points). Even though our sample had a mean of 22 months post-TBI, prior studies have successfully utilized RBANS, MoCA, and DRS in samples with similar chronicity ranges (1, 21, 57). These assessments remain valuable tools for evaluating cognitive function in individuals with chronic moderate-to-severe TBI, providing reliable and standardized measures of neurocognitive performance even in the later stages of recovery.

One participant was unable to complete the full RBANS assessment at study baseline due to cognitive fatigue, resulting in missing data for certain subtests (Language, Delayed Memory, Immediate Memory) as well as the total score. Cognitive testing was conducted by a licensed neuropsychologist or a PsyD intern under supervision of a licensed neuropsychologist.

### Statistical analysis

Statistical analyses were conducted using Jeffrey’s Amazing Statistics Program (JASP) with a *p*-value of 0.05 for significance (22). Due to the non-parametric nature of the data, Spearman’s rank-order correlation analyses were used to assess the relationships between initial neuroimaging variables with changes in cognitive scores. Prior to conducting these analyses, we tested whether age and time since injury were significantly correlated with the variables of interest. Both variables were found not to have significant correlations. However, since the heterogeneity in TSI could still potentially bias the results even without being linearly correlated with outcomes, we decided to conduct a version of the Spearman’s correlation analyses while treating TSI as a covariate. The Benjamini-Hochberg method (FDR = 0.05) was utilized for multiple comparisons correction.

Non-parametric (Wilcoxon) paired sample T-tests were conducted on RBANS, DRS, and MoCA scores in order to identify significant changes from study enrollment to 6 months.

To assess reliable change in cognitive performance over the 6-month follow-up, we calculated the Reliable Change Index (RCI) for MoCA, DRS, and each RBANS index score as well as the composite score. RCI was calculated using following formula:

To assess reliable change in cognitive performance over the 6-month follow-up, we calculated the Reliable Change Index (RCI) for MoCA, DRS, and each RBANS index score as well as the composite score. RCI was calculated using following formula:


$${\mathrm{SD}}^{*}\;\text{sqrt}{\left(2\right)}^{*}\;\text{sqrt}{\left(1-\mathrm{rel}\right)}^{*}\;\text{qnorm}(1-(1-\mathrm{CI})/2)$$

Which has emerged as a comparable approach to regression-based RCI formulas across studies of neurocognitive change in TBI populations (23, 24). In this equation, SD represents the baseline standard deviation, rel is the reliability coefficient, and CI is the 90% confidence interval (z = ±1.645), consistent with the majority of RCI literature (25, 26). For all neuropsychological measures, including RBANS composite/index scores, MoCA, and DRS, we used test–retest reliability as the reliability coefficient. Changes exceeding the RCI threshold were classified as statistically reliable improvements or declines, while changes within the threshold were not distinguishable from expected test–retest variability.

## Results

Of the 15 participants who completed initial neurocognitive assessments, 10 (67%) completed the follow-up MoCA, and 9 (60%) completed the follow-up RBANS and DRS. An attrition analysis was conducted to compare individuals who completed the study to those lost to follow-up. ANOVA results showed no significant differences between these groups in age (*p* = 0.679), time since injury (*p* = 0.587), RBANS total score (*p* = 0.539), MoCA (*p* = 0.120), or DRS (*p* = 0.227). Similarly, chi-square analyses revealed no significant differences in gender (*p* = 0.391) or ethnicity (*p* = 0.669). These results suggest that attrition was not systematically biased by demographic, injury severity, or cognitive factors.

**Figure 2:**
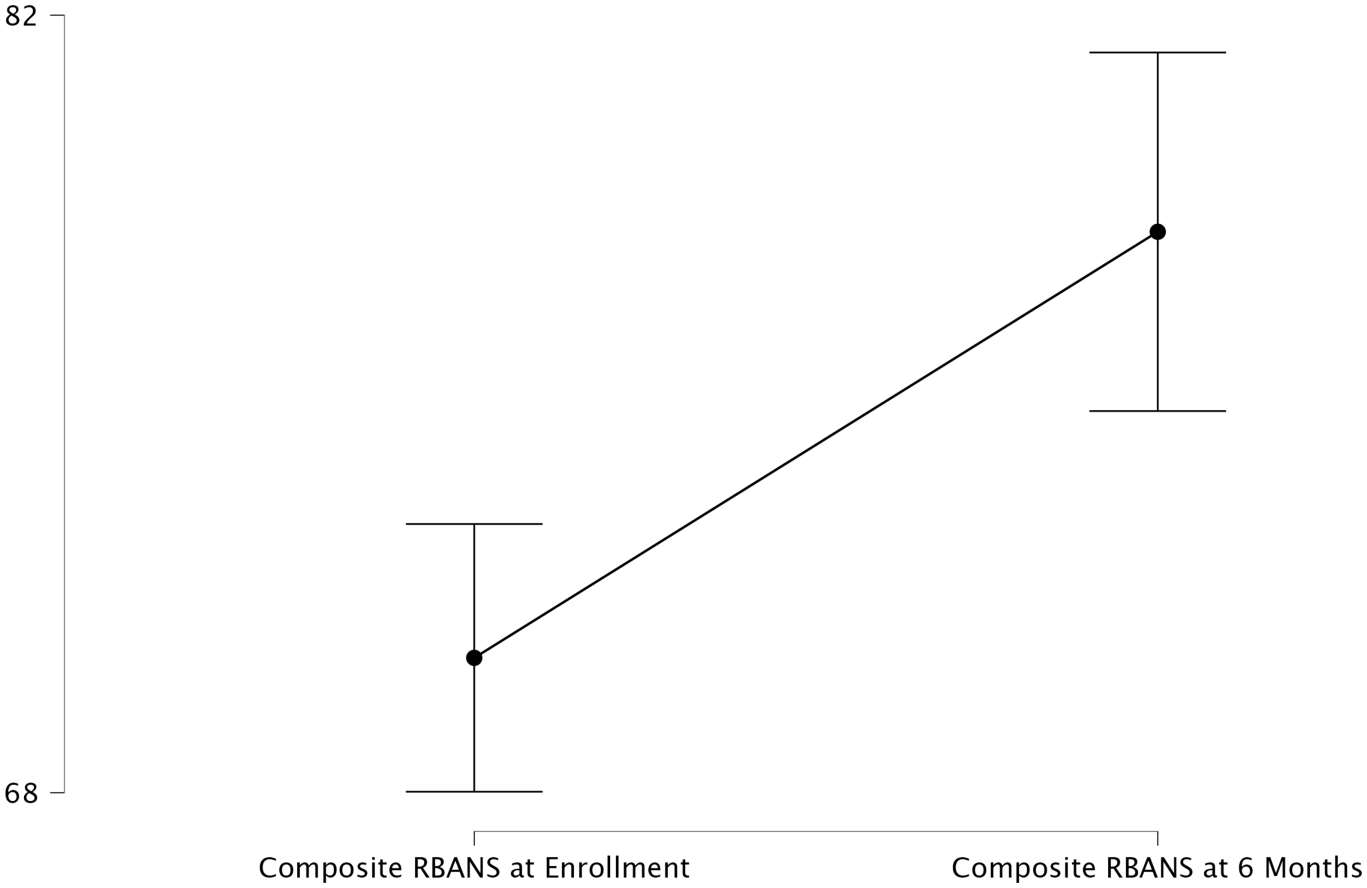
Change in composite RBANS. Change in composite RBANS score from study enrollment to six-month follow up with 95% confidence interval.

RBANS showed a significant increase from enrollment to 6 month follow up (*p* = 0.035; Figure 2), however there was no significant change in MoCA or DRS. Refer to Table 2 for specific information regarding cognitive scores at both timepoints.

**Table 2:** Enrollment and 6-month cognitive scores.

Variable	Enrollment mean (SD)	6 Month mean (SD)
Montreal Cognitive Assessment (MoCA)	21.27 (4.33), *n* = 15	22.33 (3.65), *n* = 10
Disability Rating Scale (DRS)	3.67 (2.58), *n* = 15	2.9 (2.08), *n* = 9
Repeatable Battery for the Assessment of Neuropsychological Status (RBANS)—Composite	70.429 (14.40), *n* = 14^*^	78.10 (16.67), *n* = 9^*^

* Significant change from pre to post.

RCI analysis revealed considerable individual variability in the cognitive changes observed for participants who had 6-month follow up scores (Table 3). For the RBANS Immediate Memory Index, 1 participant (11.1%) showed reliable improvement, while 1 participant (11.1%) showed a reliable decline. In the RBANS Visuospatial/Constructional Index, 1 participant (10%) demonstrated a reliable increase. Reliable improvement was observed in the RBANS Language Index for 3 participants (33.3%) and in the RBANS Attention Index for 1(10%). The RBANS Delayed Memory subtest showed a reliable decline in 2 participants (22.2%) and a reliable increase in 1 (11.1%). For the composite RBANS score, 1 participant (11.1%) showed reliable improvement, and 1 (11.1%) showed a reliable decrease. Among the MoCA scores, 1 participant (10%) showed a reliable increase, while the DRS showed a reliable decline in 1 participant (11.1%). These results show the significant variability in cognitive changes following TBI, highlighting the importance of individualized assessments. These RCI results suggest that although the group as a whole showed significant improvement in composite RBANS score, individual responses varied, with some participants showing reliable cognitive improvements, while others exhibited stable performance or declines.

**Table 3:** Neuropsychological test scores and reliable change index (RCI) thresholds.

Score	Study enrollment mean (SD)	6 Month mean (SD)	RCI	Increased (%)	Unchanged (%)	Declined (%)
RBANS Composite	70.43 (14.40)	78.10 (16.67)	9.47	1 (11.1%)	7 (77.8%)	1 (11.1%)
RBANS Immediate Memory	72.71 (13.53)	79.1 (20.19)	18.35	1 (11.1%)	7 (77.8%)	1 (11.1%)
RBANS Visuospatial/Constructional	80.73 (16.34)	85.40 (15.31)	22.65	1 (10%)	9 (90%)	0 (0%)
RBANS Language	77.29 (15.29)	91.90 (10.06)	18.82	3 (33.3%)	6 (66.7%)	0 (0%)
RBANS Attention	80.93 (17.03)	81.50 (14.691)	20.59	1 (10%)	9 (90%)	0 (0%)
RBANS Delayed Memory	69.79 (19.14)	75.80 (27.37)	16.36	1 (11.1%)	6 (67.7%)	2 (22.2%)
MoCA	21.27 (4.33)	22.33 (3.65)	3.96	1 (10%)	9 (90%)	0 (0%)
DRS	3.67 (2.58)	2.9 (2.08)	3.64	0 (0%)	9 (89.9%)	1 (11.1%)

**Figure 3:**
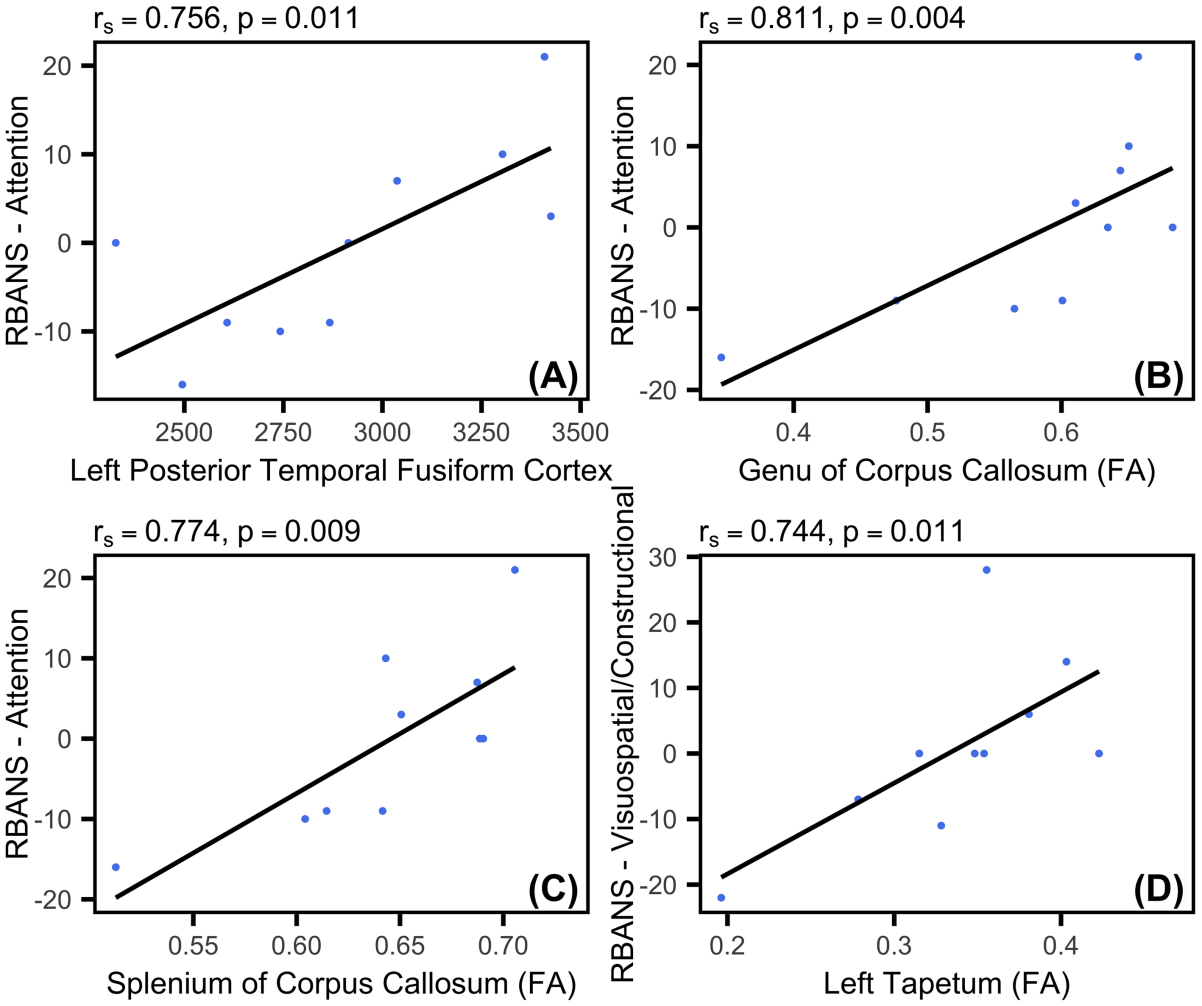
Correlation scatterplots. **(A–D)** Scatterplots indicating the Spearman’s correlation between neuroimaging variables at study enrollment and changes in RBANS index scores from study enrollment to 6 month follow up (*N* = 10). Plots are shown for **(A)** Left Posterior Temporal Fusiform Cortex (grey matter volume), **(B)** Genu of Corpus Callosum (average fractional anisotropy), **(C)** Splenium of Corpus Callosum (average fractional anisotropy), and **(D)** Left Tapetum (average fractional anisotropy). Each data point represents a participant.

After applying multiple comparisons corrections, a significant correlation was found between the initial grey matter volume of the posterior division of the left temporal fusiform cortex and 6 month change in the RBANS—Attention index score (r_s_ = 0.756 *p* = 0.011). Initial FA in corpus callosum white matter tracts also had significant associations with cognitive outcomes. Specifically, change in RBANS—Attention was correlated with study-baseline FA in the genu of the corpus callosum (r_s_ = 0.811, *p* = 0.004) as well as the splenium of the corpus callosum (r_s_ = 0.774, *p* = 0.009). FA in the left tapetum was correlated with change in the RBANS—Visuospatial/Constructional index score (r_s_ = 0.744, *p* = 0.011). See Figure 3 for scatterplots of significant correlations.

When covarying out TSI in the correlation analyses, we obtained slightly different results. Change in RBANS—Attention was correlated with study-baseline FA in the genu (r_s_ = 0.961, *p* < 0.001) and splenium of corpus callosum (r_s_ = 0.859, *p* = 0.003). FA in the left tapetum remained significantly correlated with change in RBANS—Visuospatial/Constructional (r_s_ = 0.743, *p* = 0.022). However, the left temporal fusiform cortex correlation with RBANS—Attention was no longer significant (r_s_ = 0.593, *p* = 0.092).

## Discussion

Our study aimed to explore the various biomarkers of recovery from traumatic brain injury by investigating the relationship between variables at study baseline and change in cognitive scores over a 6-month period. It is important to note that our study was designed as an exploratory investigation into the prognostic value of neuroimaging markers for cognitive change and recovery in TBI. Additionally, given our small sample size, findings should not be interpreted as definitive but rather as preliminary associations that can help guide future research. Our findings can be summarized as follows: (1) There was significant change in RBANS from study enrollment to 6-month follow up; (2) Initial FA in temporal and interhemispheric white matter tracts were positively correlated with changes in various cognitive scores; (3) Initial grey matter volume in the left temporal fusiform cortex was positively correlated with improvements in attention.

While no significant changes were observed between study enrollment and follow-up cognitive domain scores on DRS and MoCA, composite RBANS showed significant improvements over the 6-month period. This highlights the possibly greater sensitivity and comprehensiveness of the RBANS compared to both the MoCA and DRS in assessing cognitive recovery following TBI. The MoCA, while valuable as a screening tool for global cognitive function, and the DRS, which primarily measures disability rather than cognitive performance, may not capture subtle changes in specific domains as effectively as the RBANS (27, 28). Additionally, despite the high variability in time since injury among participants, the significant improvement observed in RBANS suggests that cognitive recovery still seems to occur beyond the early critical recovery period (29, 30). RCI analysis revealed individual variability in the cognitive changes across participants, as varying increases and decreases were seen in the MoCA, DRS, and RBANS index/composite scores. These findings emphasize the importance of individualized assessments, as some participants showed improvements, others demonstrated declines, and many remained stable. Despite significant improvement in certain cognitive scores (as seen in the t-tests) and lack thereof in others, the RCI analysis shows that cognitive change following TBI is highly individualized. This underscores the need for personalized approaches in assessing cognitive change, as group-level analyses may not fully capture the complexity of recovery. Tailored assessments that track each participant’s unique cognitive trajectory could lead to more precise insights and improve intervention strategies.

The wide variability in time since injury to study enrollment (mean = 22.27 months, SD = 39.74) presents both limitations and strengths in interpreting our findings. Given that cognitive recovery following TBI follows a nonlinear trajectory, with the most rapid improvements occurring within the first year post-injury, it is possible that some participants, particularly those with moderate TBI, had already reached a plateau in their recovery by the time of their first assessment (29–31). This may have contributed to the lack of significant changes in MoCA and DRS scores between study enrollment and follow-up. Despite this however, significant correlations between neuroimaging measures at study baseline and changes in RBANS scores were still observed, suggesting that structural brain characteristics at the time of study enrollment remained associated with cognitive change, even beyond the early post-injury period. This finding emphasizes the relevance of neuroimaging biomarkers in potentially identifying residual structural integrity following TBI that may continue to shape change in cognitive function. Additionally, while early post-injury assessments are critical for tracking dynamic recovery, studying individuals later in their recovery trajectory possibly provides insights into the long-term stability of brain-behavior relationships as well as the potential for structural markers to serve as correlates of cognitive improvement.

In assessing the structural volume associations with cognitive change, the temporal fusiform cortex is the outer layer of grey matter on the fusiform gyrus that is located on the temporal lobe (32). It is strategically positioned within the ventral visual stream, which allows it to serve as a key structure in high-level visual processing. It also functions in object recognition and the processing of complex visual stimuli such as faces (33–35). Higher grey matter volume in this region could positively contribute to change in RBANS—Attention index score in several ways. First, the temporal fusiform cortex seems to have a direct relationship with attentive ability via the aforementioned visual processing. Increased grey matter in the region could also indicate better structural preservation of neural areas critical for visual processing and attention, which could provide a stronger foundation for recovery over time (36). Studies have also shown that neuroplasticity can be enhanced via increased use of relevant brain regions—indicating a possibility of heightened attentive ability leading to increased improvements in attention (2, 3, 37).

Our study also found that FA of certain white matter tracts correlated with improvements in cognitive scores over 6 months. FA has been found to be a proxy for the integrity of white matter microstructures such as axons and myelin, with lower levels of FA usually being associated with worse cognitive and behavioral outcomes (7). The significant correlations between initial FA in the genu and splenium of corpus callosum with the RBANS—Attention index score potentially underscore the importance of white matter microstructure in cognitive recovery following TBI. The genu, consisting of white matter fibers that connect the prefrontal cortices, is involved in executive functions like attentional control and working memory (38–40). The splenium connects the temporal and occipital regions of both hemispheres, playing a large role in the transfer of visuospatial processing and sensory information between lobes (38, 41, 42). The increased FA in these two areas is associated with improvements in attention-based cognitive function, potentially due to facilitation of more efficient interhemispheric information transfer. It could also support better top-down control of attention usage in regards to visuospatial tasks, which could result in faster and more accurate shifting between stimuli. This would reflect more cognitive flexibility in neuropsychological testing. The improved efficient interhemispheric communication from these regions could improve the redistribution of cognitive load across both hemispheres, potentially enhancing capacity for adaptive reorganization following TBI as well (43–47).

The left tapetum, a white matter tract that runs along the lateral aspect of the corpus callosum adjacent to the optic radiation, was associated with improvements in the RBANS—Visuospatial/Constructional index score. This association is likely due to its anatomy as it contains fibers from both the splenium and body of corpus callosum (48). It may play a role in visual processing similar to that of the splenium, as well as contribute to interhemispheric processing for visual and spatial information. Alternatively, this correlation could be caused by the challenges of delineating the tapetum from the optic radiations. As registration of the JHU white matter atlas between standard MNI space and subjects’ native diffusion space was necessary for delineation, inaccuracies may have been introduced into the measurements. This process could have potentially led to slight misalignments in the delineation of closely adjacent structures (49, 50). Consequently, it is possible that the ROI used to measure the tapetum’s FA may have inadvertently included some of the white matter voxels from the optic radiation. The optic radiation is crucial for the transmission of visual information from the thalamus to the visual cortex, so it could provide a plausible explanation for the observed correlation with visuospatial/constructional index scores (51, 52). Future exploration should look to disentangle the contributions of the optic radiation and tapetum in visuospatial recovery.

We note that when conducting correlation analyses while including TSI as a covariate, the correlations in white matter integrity got stronger or remained, while the correlation in grey matter volume became non-significant. This divergence is consistent with prior work that has shown that atrophy trajectories after TBI are temporally selective, with cortical and deep grey matter loss predominating a different timeframe post-injury compared to white matter atrophy (53, 54). Major commissural tracts such as the genu or splenium of corpus callosum are among the most vulnerable structures in TBI, and microstructural disruption of cortical and cerebellar white matter has been repeatedly linked to cognitive outcomes (55, 56). Taken together, these findings suggest that white matter integrity may represent a more robust and stable marker of cognitive outcome following TBI. Whereas gray matter volume may have weaker or more time-sensitive associations—potentially explaining why the gray matter effect attenuated after adjusting for TSI (53, 54).

## Limitations

This study is subject to certain limitations. Detailed ethnoracial data was not collected, limiting our ability to examine the potential impact of racial and ethnic background on cognitive recovery. Additionally, although all participants were fluent English speakers, information on whether English was their primary language was not gathered, which may be relevant to interpreting cognitive test performance. Furthermore, since neurocognitive assessments were conducted within the same session at each time point, there is a possibility of training effects or test contamination, where prior exposure to similar cognitive tasks could influence subsequent performance. Future studies should include comprehensive demographic data and consider separating cognitive assessments across different sessions to mitigate potential practice effects and ensure independent measurement of cognitive domains.

A key limitation of this study is the limited assessment of executive function and working memory within the RBANS. While RBANS provides a broad evaluation of cognitive domains, it lacks direct measures of working memory (e.g., digit span backwards), response inhibition, cognitive set-shifting, or problem-solving abilities (e.g., maze tasks)—all of which are frequently impaired in TBI. Given that executive dysfunction is a hallmark of TBI, certain deficits may not have been adequately captured. Though the cognitive profile of a TBI patient varies based on the nature of injury, future studies should also target executive function and working memory to provide a more complete understanding of cognitive recovery trajectories.

The study’s focus on specific regions of interest, while informative, could overlook other critical regions involved in TBI recovery. There is also a lack of exploration of the subcortical brain regions. Future research should aim to explore a broader range of brain regions and incorporate more specific delineation of the optic radiation and tapetum in order to provide a more complete picture of the changes following TBI. Additionally, the exclusion of other scalar diffusion imaging metrics such as mean diffusivity, radial diffusivity, etc. limits our ability to understand microstructural mechanisms of cognitive recovery. Future studies should include these in their analyses in order to gain insight into axonal and myelin damage, which could further elucidate the role of white matter in post-TBI recovery trajectories. Another limitation is the clinical availability of neuroimaging techniques. While T1-weighted MRI is standard in acute TBI assessment, DTI is not routinely used, and would be an additional cost and resource constraint, limiting its feasibility for widespread clinical application.

Furthermore, the study’s focus on 6 months post study enrollment may not overlap with the full trajectory of cognitive recovery, which can continue for years following TBI (30, 31). Each participant joined the study at different lengths of time following their TBI onset, leading to considerable variability in time since injury, as reflected in the large standard deviation (SD = 39.74 months). This could have confounded the analysis, as recovery trajectories may differ at critical periods following injury (29, 30). A long-term follow up coupled with a consistent time from injury to study enrollment could provide more stability of observed associations. Additionally, cognitive rehabilitation status was not systematically assessed, which could influence recovery trajectories. Variability in prior rehabilitation may have impacted cognitive outcomes, making it a potential confounder. However, this heterogeneity may be informative. The observation of significant correlations despite such variability in TSI suggests that associations may not be limited to a narrow recovery window. Replication in larger and more stratified cohorts will be required, but this raises the possibility that these relationships exist even when assessed at widely varying times post-TBI.

A final limitation of this study is the small final follow-up sample size (n = 9–10) due to participant attrition. While attrition analysis revealed no significant differences between individuals who completed the study and those lost to follow-up in terms of demographics, injury severity, or baseline cognitive performance, the reduced sample size limits statistical power and the generalizability of the findings. Additionally, our small sample size yields a higher risk of both Type I and Type II errors. Studies with small neuroimaging and cognitive outcome datasets also face challenges in replication. Future research should prioritize larger sample sizes and improved retention strategies, such as remote follow-ups, participant incentives, and flexible scheduling to enhance study reproducibility and statistical reliability. They should also look to include a healthy control cohort, as well as mild TBI cases, in order to strengthen the generalizability of associations.

## Conclusion

This study offers valuable insights into the role of white matter integrity and grey matter volume in cognitive recovery following moderate to severe TBI. Our findings suggest that neuroimaging markers within interhemispheric tracts and temporal regions may have some clinical relevance to the trajectory of cognitive recovery. The observed associations highlight the potential of these markers to serve as indicators of neuroplasticity and recovery, and also encourage their integration into personalized rehabilitation strategies. We hope that through this preliminary exploratory study, we can help inform future power analyses and also save resources in future studies by focusing attention on more promising markers. Future research should aim to incorporate additional neuroimaging modalities and examine a broader range of brain regions to better characterize potential biomarkers, ultimately contributing to a more comprehensive understanding of long-term recovery in TBI patients.

## Data Availability

The raw data supporting the conclusions of this article will be made available by the authors, without undue reservation.

## Footnotes

^1^ https://www.nitrc.org/projects/dtiprep/
